# The Lazarus *Escherichia coli* Effect: Recovery of Productivity on Glycerol/Lactose Mixed Feed in Continuous Biomanufacturing

**DOI:** 10.3389/fbioe.2020.00993

**Published:** 2020-08-13

**Authors:** Stefan Kittler, Julian Kopp, Patrick Gwen Veelenturf, Oliver Spadiut, Frank Delvigne, Christoph Herwig, Christoph Slouka

**Affiliations:** ^1^Research Division Biochemical Engineering, Research Group Integrated Bioprocess Development, Institute of Chemical, Environmental and Bioscience Engineering, Vienna University of Technology, Vienna, Austria; ^2^Christian Doppler Laboratory for Mechanistic and Physiological Methods for Improved Bioprocesses, Institute of Chemical, Environmental and Bioscience Engineering, TU Vienna, Vienna, Austria; ^3^TERRA Teaching and Research Centre, Microbial Processes and Interactions (MiPI), Gembloux Agro-Bio Tech – Université de Liège, Gembloux, Belgium

**Keywords:** *E. coli*, recombinant protein production, continuous biomanufacturing, mixed-feeding, productivity recovery

## Abstract

Continuous cultivation with *Escherichia coli* has several benefits compared to classical fed-batch cultivation. The economic benefits would be a stable process, which leads to time independent quality of the product, and hence ease the downstream process. However, continuous biomanufacturing with *E. coli* is known to exhibit a drop of productivity after about 4–5 days of cultivation depending on dilution rate. These cultivations are generally performed on glucose, being the favorite carbon source for *E. coli* and used in combination with isopropyl β-D-1 thiogalactopyranoside (IPTG) for induction. In recent works, harsh induction with IPTG was changed to softer induction using lactose for T7-based plasmids, with the result of reducing the metabolic stress and tunability of productivity. These mixed feed systems based on glucose and lactose result in high amounts of correctly folded protein. In this study we used different mixed feed systems with glucose/lactose and glycerol/lactose to investigate productivity of *E. coli* based chemostats. We tested different strains producing three model proteins, with the final aim of a stable long-time protein expression. While glucose fed chemostats showed the well-known drop in productivity after a certain process time, glycerol fed cultivations recovered productivity after about 150 h of induction, which corresponds to around 30 generation times. We want to further highlight that the cellular response upon galactose utilization in *E. coli* BL21(DE3), might be causing fluctuating productivity, as galactose is referred to be a weak inducer. This “Lazarus” phenomenon has not been described in literature before and may enable a stabilization of continuous cultivation with *E. coli* using different carbon sources.

## Introduction

The gram-negative bacterium *Escherichia coli* is the expression host of choice to produce 30% to 40% of recombinant enzymes [recombinant protein production (RPP)] in industry (Walsh, 2010; Gupta and Shukla, 2016). This organism has fast replication rates and can be cultivated on comparatively cheap media (DeLisa et al., 1999) and has high intracellular product titers. These benefits often outweigh the numerous purification steps (Berlec and Strukelj, 2013; Gupta and Shukla, 2016) and the missing glycosylation pattern on the recombinant product (Spadiut et al., 2014; Baeshen et al., 2015). The strain BL21(DE3) generated by Studier and Moffatt (1986) is often used in industrial scale, because of showing low acetate formation, high replication rates as an effect of the integrated T7-polymerase (Steen et al., 1986; Studier and Moffatt, 1986; Studier et al., 1990; Dubendorff and Studier, 1991; Neubauer and Hofmann, 1994; Lyakhov et al., 1998), as well as the possibility of protein secretion into the fermentation broth due to a type 2 secretion pathway (Jeong et al., 2009, 2015; Tseng et al., 2009). The *lac* operon is still one of the most favored promotors in pET-expression-systems (Dubendorff and Studier, 1991; Marbach and Bettenbrock, 2012; Wurm et al., 2016), therefore it is generally used for insertion of the gene of interest. Induction can be performed by addition of lactose or a structural analog, e.g., the well-known inducer isopropyl β-D-1 thiogalactopyranoside (IPTG) (Burstein et al., 1965; Neubauer and Hofmann, 1994; Wurm et al., 2016). IPTG is known to bind directly to the *lacI* repressor protein after uptake, whereas lactose needs to be transformed to allolactose to cause the blockage of the repressor protein (Burstein et al., 1965). Still, these “low-cost” products in *E. coli* with the pET system, are expensive in their making (Jia and Jeon, 2016), as one gram of IPTG can exceed the price of one gram of 900 gold. For economic reasons and for reduction of metabolic/product burden (Malakar and Venkatesh, 2012), lactose, generally regarded as waste product, can be used for induction (Wurm et al., 2016, 2018; Wurm et al., 2017a). Apart from induction mechanism, a replacement of the primary carbon source is also frequently discussed. The most favored carbon source in *E. coli* cultivations has always been glucose (Postma et al., 1993; Ronimus and Morgan, 2003; Deutscher et al., 2006). However, compared to other carbon sources glucose is quite expensive and causes diauxic growth upon lactose induction (Monod, 1949; Loomis and Magasanik, 1967). Glycerol has shown quite promising results in terms of biomass/substrate yield in *E. coli* cultivations (Blommel et al., 2007). In addition, mixtures of glucose, glycerol and lactose have shown good results for diverse products gained via autoinduction systems (Monod, 1949; Viitanen et al., 2003; Blommel et al., 2007). Recently, we presented that glycerol used as primary carbon source for *E. coli* cultivations performed equally well during biomass production as glucose, but even increased the specific product titer in fed batches (Kopp et al., 2017).

Most processes today rely on fed batch mode, which are state-of-the art in industry. They can be highly affected by different process parameters, like pH and T, physiological feeding (adaption of the specific substrate uptake rate) and change in induction agent. However, the time dependence of product quality is still a major drawback using this cultivation technique. This makes determination of the correct harvest time point often challenging, as the intracellular stress often leads to very quick lysis of the cells and product degradation. This results in variations in the downstream purification process. Continuous biomanufacturing is considered to increase product quality and therefore to a facilitation of the downstream process. However, up to now, only one stable microbial industrial chemostat process was established for *Saccharomyces cerevisiae* in the 90’s for production of insulin (Diers et al., 1991). A drop of productivity after a certain process time and the lower time-space yield hinder continuous upstream using microbial hosts from being applied in industry (Peebo and Neubauer, 2018; Kopp et al., 2019b).

Several efforts have been made to enable continuous processes in *E. coli* (Kopp et al., 2019a), but we are still far away from application of such systems. Long-term cultivations with *E. coli* showed enhanced cell burden using IPTG induction and clearly favored feeding of the disaccharide lactose (Dvorak et al., 2015). From an engineering point of view, higher growth rates seem to be beneficial for RPP as shown in chemostat- and fed-batch experiments (Vaiphei et al., 2009; Slouka et al., 2019). Mutations and plasmid loss are expected during long time cultivation of *E. coli* (Weikert et al., 1997; Sieben et al., 2016; Peebo and Neubauer, 2018). However, constant supply of antibiotics is believed to prevent plasmid loss in continuous cultures (Sieben et al., 2016). Change in gene regulation of the transcription upon long time cultivation are often reported. The *lac* operon resulting in multistability of induction is well known and reported in literature (Ozbudak et al., 2004). Fluctuations in plasmid number, lac-repressor and cAMP levels may drastically influence RPP during long time cultivation. High concentrations of metabolites, like galactose upon feeding of lactose, are known to affect β-galactosidase concentrations (Llanes and McFall, 1969; Portaccio et al., 1998). Other approaches try to integrate the gene of interest into the genome to receive stable production in combination with strong inducible promotors (Striedner et al., 2010). Reactor design is a further screw to overcome stability issues in long-term RPP with *E. coli*, as recently published by Schmideder and Weuster-Botz (2017) who implemented a cascaded system, where two stirred tank reactors are operated in parallel at different conditions.

In this contribution, we present the results of chemostat cultures using mixed feed systems first applied by Wurm et al. (2016, 2018). The goal was to accomplish a continuous process with stable productivity outperforming the frequently used fed-batch. We tested mixed feeds with glucose/lactose and glycerol/lactose in chemostat for three model proteins and compared the performance to state-of-the-art fed batches induced with IPTG. In contrast to cultivations using BL21(DE3) with glucose/lactose, glycerol/lactose-based cultivations showed a recovery of productivity at elevated induction time. Alterations regarding the choice of carbon source thus might be a key driver to possible enable stable productivity on a long-term basis. As an already lost productivity is recovered, we annotated this resurrection of productivity with the Christian term of “Lazarus” and the corresponding “Lazarus effect.”

## Materials and Methods

### Strains

Cultivations were carried out with the strain *E. coli* Bl21(DE3) using three different model proteins. All three protein sequences were cloned in a pET vectors, using pET21a^+^ for green fluorescent protein (GFP), and pET28a for mCherry protein (mCherry) and Blitzenblue (BBlue). mCherry and BBlue were thankfully received from Prof. Maurer at FH Campus Wien. All cultures were kept at −80°C in 25% glycerol cryo stocks. The extracted pET21a^+^ plasmid encoding for GFP was extracted and electroporated into an HMS174(DE3) strain (Novagen, Merck, Darmstadt, Germany).

### Cultivation and Process Modes

Cultivations were executed in a Minifors 2 bioreactor system (max. working volume: 1 L; Infors HT, Bottmingen, Switzerland). All cultivations were carried out using a defined minimal medium referred to DeLisa et al. (1999). Media had the same composition with different amounts of glycerol and glucose. Details on pre-culture, batch, fed batch and chemostat cultivation are given in Table 1. Induction was performed according to Table 1. The ratio of glycerol to lactose was calculated based on recent works in fed-batch regarding maximal lactose uptake versus applied q_s,C_ (Wurm et al., 2016). Fed batch was always cultivated to have a standard for protein production with similar specific growth rate as applied in the chemostat. The cultivation offgas flow was analyzed online using gas sensors – IR for CO_2_ and ZrO_2_ based for Oxygen (BlueSens Gas analytics, Herten, Germany). Process control and feeding was established using the process control system PIMS Lucullus (Securecell, Urdorf, Switzerland).

**TABLE 1:** C source and induction for the different cultivations.

	Glucose [g/L]	Glycerol [g/L]

Pre-culture media	8	8
Batch media	20	20

Feed	Glucose [g/L]	Glycerol [g/L]	Inducer	Inducer concentration
GFP chemostat mode 1		50	Lactose	25 g/L
GFP chemostat mode 2	50		Lactose	25 g/L
GFP Fed-batch mode	300		IPTG	0.5 mM
mCherry chemostat mode		50	Lactose	25 g/L
mCherry Fed-batch mode	300		IPTG	0.5 mM
BBlue chemostat mode		50	Lactose	25 g/L
BBlue Fed-batch mode	400		IPTG	0.5 mM
HMS GFP chemostat 1		50	Lactose	25 g/L
HMS GFP chemostat 2	50		Lactose	25 g/L

We used glucose or glycerol as carbon source and 0.5 mM IPTG for induction. During all induction phases pH is kept constant at 6.7 and temperature at 30°C. pH was controlled with base only (12.5% NH_4_OH), while acid (10% H_3_PO_4_) was added manually, if necessary. The pH was monitored using a pH-sensor EasyFerm Plus (Hamilton, Reno, NV, United States). Aeration was carried out using a mixture of pressurized air and pure oxygen at about 2 vvm to keep dissolved oxygen (dO_2_) always higher than 30 %. The dissolved oxygen was monitored using a fluorescence dissolved oxygen electrode Visiferm DO (Hamilton, Reno, NV, United States).

For fed batches static feed forward q_s_-controls were performed during induction phase. Exponential feed was established according to Eq. 1, to keep q_s,C_ constant (Slouka et al., 2016; Wurm et al., 2016):

(1) $$F\,\left(t\right)=\frac{q_{S,C}*X\,\left(t\right)*\rho_{f}}{C_{f}}$$

With F being the feedrate [g/h], q_s,C_ the specific glycerol uptake rate [g/g/h], X(t) the absolute biomass [g], ρ_F_ the feed density [g/L], and c_F_ the feed concentration [g/L], respectively. For applied control strategies adaption of the q_s,C_ during the induction time was performed based on Eq. 1. Chemostat cultivations were set manually to a dilution rate of *D* = 0.1 h^–1^ for all performed runs.

### Process Analytics

For fed batch, samples were always taken after inoculation, upon end of the batch-phase and after the non-induced-fed batch was finished. During the induction period, samples were taken in a maximum of 120 min intervals and analyzed subsequently. Details on the process analytics can be found elsewhere (Kopp et al., 2018; Slouka et al., 2018). For chemostat cultivations samples were collected after batch and afterward once or, if necessary, twice a day.

### Product Analytics

#### Preparation

A 5 mL fermentation broth samples were centrifuged at 4800 rpm at 4°C for 10 min. The supernatant was discarded, and the pellet was resuspended to a DCW of about 4 g/L in lysis buffer (100 mM Tris, 10 mM EDTA at pH = 7.4). Afterward the sample was homogenized using a Gea PandaPlus homogenizer (Gea, AG, Germany) at 1500 bar for 10 passages. After centrifugation at 10000 rpm and 4°C for 10 min 10 ml of the supernatant were kept for analysis of the soluble protein. Soluble protein was stored in 4°C. The resulting IB pellet was washed twice with ultrapure water. Aliquoted of pellets à 2 mL broth were centrifuged again (14000 rpm, 10 min 4°C) and finally stored at −20°C.

#### Inclusion Body Titer

Soluble protein samples were filtered (0.2 μm mesh) and directly used in the HPLC. Inclusion Body (IB) pellet samples were prepared according to Kopp et al. (2018), and subsequently solubilized using following buffer: 7.5 M guanidine hydrochloride, 62 mM Tris at pH = 8 and 125 mM DTT was added right before use. The filtered IB samples were quantified by HPLC analysis (UltiMate 3000; Thermo Fisher, Waltham, MA, United States). The used column was manufactured by Waters (BioResolve RPmab) and was designed for monoclonal antibody measurements (Waters Corporation, Milford, MA, United States). The product was quantified with an UV detector (Thermo Fisher, Waltham, MA, United States) at 280 nm, respectively. Mobile phase was composed of water (ultrapure) (eluent A) and acetonitrile (eluent B) both supplemented with 0.1% (v/v) trifluoride acetic acid. Details on the method are given in Kopp et al. (2020). For quantification of soluble protein, a size exclusion (=SEC) chromatography principle was applied, using a X-bridge column (Waters Corporation, ıMilford, MA, United States). The mobile phase was composed of 250 mM KCl and 50 mM of each KH_2_PO_4_ and K_2_HPO_4_ dissolved in Ultrapure water as described elsewhere (Goyon et al., 2018). A constant flow rate of 0.5 mL/min was applied with an isocratic elution at 25°C for 18 min. BSA standards (50, 140, 225, 320, 500, 1000, and 2000 mg/mL; Sigma Aldrich, St. Louis, MO, United States) were used for quantification.

## Results

Fed batch cultivations induced with IPTG are the golden standard for RPP with *E. coli*. To be able to compete with fed batches, the specific productivity in chemostat cultivations must be maximized and the duration of the productive phase prolonged, so that time space yields are significantly increased. We tested two mixed feed systems, glucose/lactose, and glycerol/lactose, to find conditions for stable protein expression in *E. coli* chemostats.

### Fed-Batch and Chemostats Using Bl21(DE3) Expressing GFP as Model Protein

**FIGURE 1:**
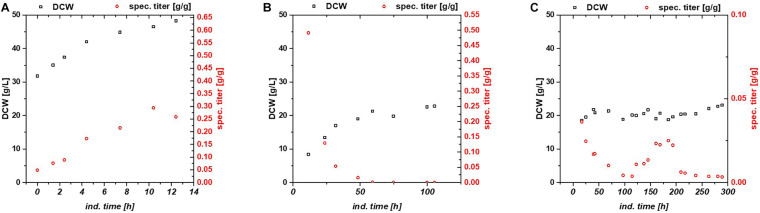
Comparison between **(A)** fed batch, **(B)** glucose/lactose fed chemostat, and **(C)** glycerol/lactose chemostat for production of recombinant GFP using Bl21(DE3).

First, we compared three different cultivation modes using GFP as model protein. As IPTG is often referred to as toxic to the cells after a certain induction time, we used lactose as mild inducer in the first run (Dvorak et al., 2015). A mixed feed consisting of glucose and lactose (Table 1) was used for the first chemostat (Figure 1). To ease the comparability between fed batch and chemostat cultivation we aimed for similar specific substrate uptake rates – q_s,C_, which resulted in a dilution rate for the chemostats of 0.1 h^–1^. Respective process parameters are presented in Table 2.

**TABLE 2:** Process variables for three performed cultivations with Bl21(DE3) expressing GFP.

Process parameters	Glucose-IPTG fed-batch	Glucose-lactose chemostat	Glycerol-lactose chemostat
spec. titer max. [g/g]	0.30	0.31	0.04
q_p,max_ [g/g/h]	0.05	0.01	0.01
q_s,C_ [g/g/h]	0.23 ± 0.02	0.24 ± 0.04	0.27 ± 0.04
Y_X/S_ [g/g]	0.39 ± 0.09	0.49 ± 0.09	0.42 ± 0.03

While the fed batch gave the highest product titers after 10 h of induction, the glucose/lactose chemostat system had the maximum product titer at 20 h. In the glucose chemostat, productivity dropped below the limit of quantification (LoQ) of the HPLC method after around 3 days of cultivation, including batch of 6 h, and did not recover at later induction times. This phenomenon is well known for other strains cultivated with glucose as sole carbon source in chemostat mode (Peebo and Neubauer, 2018). Based on our recent results in fed batch using glycerol as carbon source (Kopp et al., 2017), we cultivated *E. coli* producing GFP with a mixed feed of glycerol/lactose. Highest specific titer (calculated by dividing titer through biomass), was found in the beginning of the glucose/lactose chemostat (Figure 1). There are high differences in terms of productivity between the three cultivations (Table 2).

In general, inclusion bodies and soluble protein were produced simultaneously during cultivations of *E. coli* expressing GFP (Wurm et al., 2018). For easy comparison, we calculated the total productivity as a sum of IBs and soluble protein. Productivity and titer on glycerol were far lower in the beginning in comparison to the glucose/lactose fed chemostat (compare axes in Figure 1). In contrast to the glucose/lactose chemostat a recovery in productivity appeared at about 150 h of induction time. This renewed productivity dropped again at about 250 h of induction.

### Chemostat Cultivation Using *E. coli* HMS174 Expressing GFP

**FIGURE 2:**
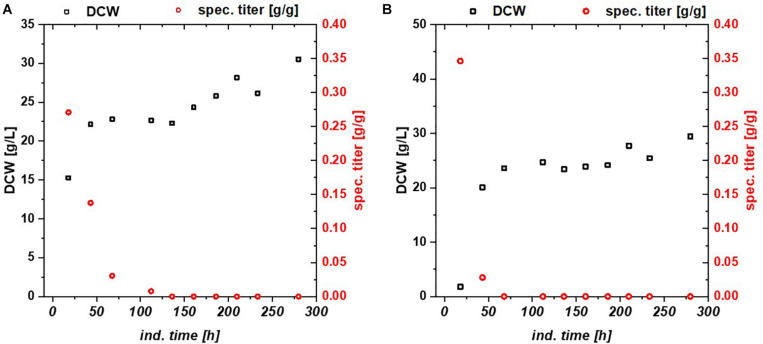
HMS174 expressing GFP **(A)** using a glucose/lactose mixed feed and **(B)** a glycerol/lactose mixed feed. In neither of the cultivations a recovery of productivity is observed. Based on consumption of galactose, the overall DCW is higher in both cultivations.

*Escherichia coli* Bl21(DE3) lacks in a functioning Leloir pathway implementing lacking galactose uptake (Daegelen et al., 2009). To investigate effects of galactose metabolism, a recombinant *E. coli* HSM174(DE3) strain with a working galactose metabolism was examined. *E. coli* HSM174(DE3) was cultivated with glycerol and glucose as primary carbon source and lactose as inducer in chemostat cultures (Table 3). In Figure 2A, the chemostat cultivation with glucose/lactose is shown. HMS strain behaves like Bl21 with initial high productivity of GFP expressed in similar specific titers. Biomass concentration was in overall higher using this strain, as galactose served as additional carbon source. The glycerol based chemostat, given in Figure 2B, showed also comparable productivity after induction to Bl21, however, no recovery after 100 h of induction was observed using HMS174(DE3) strain.

**TABLE 3:** Process variables for performed chemostat cultivations with HMS174(DE3) expressing GFP.

Process parameters	Glucose-lactose chemostat	Glycerol-lactose chemostat
spec. titer max. [g/g]	0.27	0.35
q_p,max_ [g/g/h]	0.02	0.039
q_s,C_ [g/g/h]	0.27 ± 0.05	0.27 ± 0.02
Y_X/S_ [g/g]	0.37 ± 0.04	0.43 ± 0.03

### Chemostat Cultivation Using Bl21(DE3) Expressing mCherry and Blitzenblue as Model Proteins

To check the effects of glycerol/lactose chemostats, we produced two further fluorescent model proteins with this respective system and compared them to glucose/IPTG fed batches using the strain Bl21(DE3). In Table 4 results for cultivation using *E. coli* expressing mCherry are presented. Higher productivity and specific titer were found for the fed batch cultivations. However, induction with IPTG and product titers up to 18 g/L imposed stress to cells, obvious in the low substrate to biomass yield.

**TABLE 4:** Cultivation variables for fed batch cultivation and continuous cultivation with the glycerol/lactose system for production of recombinant mCherry and recombinant Blitzenblue.

	Glucose-IPTG fed-batch	Glycerol-lactose chemostat
**Process variables mCherry**		
spec. titer max [g/g]	0.21	0.17
q_p,max_ [g/g/h]	0.069	0.01
q_s,C_ [g/g/h]	0.28 ± 0.02	0.22 ± 0.2
Y_X/S_ [g/g]	0.34 ± 0.09	0.47 ± 0.04
**Process variables BBlue**		
spec. titer max [g/g]	0.13	0.035
q_p,max_ [g/g/h]	0.033	0.002
q_s,C_ [g/g/h]	0.29 ± 0.01	0.25 ± 0.05
Y_X/S_ [g/g]	0.18 ± 0.05	0.30 ± 0.03

A similar result was obtained for cultivation for *E. coli* recombinantly expressing BBlue (Table 4). The magnitude of specific titer and q_p,max_ differed, but the trends were comparable. The substrate to biomass yield was even lower than for mCherry indicating that BBlue even imposed more stress to the host in the fed batch mode.

**FIGURE 3:**
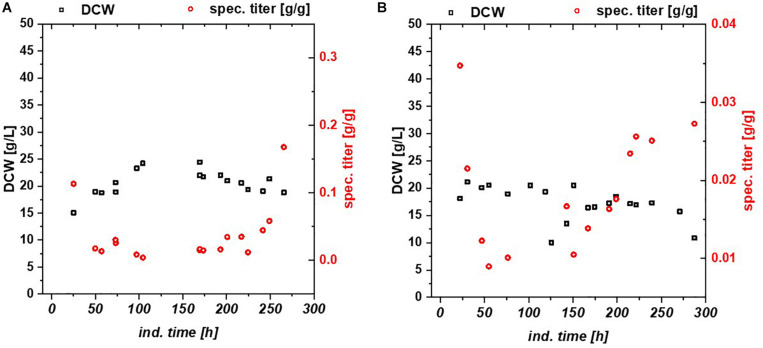
Chemostat cultures with Bl21(DE3) with glycerol/lactose mixed feed expressing **(A)** mCherry, **(B)** Blitzenblue. The recovery of the productivity is clearly visible at 200 h in both cultivations. Blitzenblue **(B)** exhibits only soluble protein in fed batch and continuous cultivation.

Chemostats for mCherry and BBlue resulted in less cellular stress compared to the fed batch cultivation. In Figure 3 DCW, specific titer and IB fraction over time are plotted for mCherry (Figure 3A) and BBlue (Figure 3B). mCherry produced a high amount of IBs in the beginning of the chemostat upon the first decrease in productivity during the cultivation. The recovery of productivity at about 200 h was exclusively soluble protein with no IB fraction anymore. BBlue showed no IB expression in fed batch nor in the chemostat and a high specific titer at the recovery at 200 h of induction time. This effect was highly different to the GFP glycerol run. While a high IB ratio was present in the GFP glycerol chemostat, the other *E. coli* expressing mCherry and BBlue produced mainly soluble protein in the recovery phase. During intermediate time between 100 and 200 h biomass concentration was generally higher (also indicated by higher yield coefficients), so metabolism was most likely regulated toward anabolism of host cell proteins. During the recovery, DCW started to drop again because of recombinant protein expression. In contrast to GFP and BBlue, mCherry had a high overall specific titer within the entire cultivation. Elucidating this metabolic shift in regulation may be a key parameter for maintaining constant productivity in chemostat cultures.

## Discussion

**FIGURE 4:**
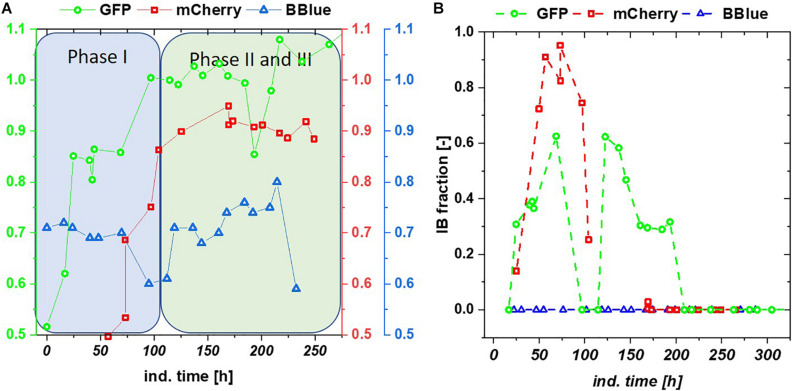
**(A)** C-balances of all three chemostat cultivations with glycerol/lactose feed. Blitzenblue does not reach a balance of 1 in the entire cultivation time and does not have adaption phase I. **(B)** Inclusion body (IB) fractions over the timespan of glycerol-lactose chemostat cultivations: GFP IB amount increased up until 100 h and followed a subsequent drop. A recovery after 125 h of the IB fraction could be observed, mCherry showed higher IB content in the beginning of the cultivation but only recovered in productivity of the soluble fraction whereas the IB formation stopped after 100 h. Blitzenblue showed no IB-formation.

We tested the effects on productivity using mixed feed systems with glucose/lactose and glycerol/lactose in chemostats and compared them to classical fed batches with similar specific uptake rates. In the performed chemostat cultivation, we could distinguish between three different metabolic states (Figure 4A). Phase I is the adaption phase after the batch. This phase resembled a fed batch like behavior for *E. coli* Bl21(DE3) with GFP and mCherry including high RPP. This effect was most likely linked to high IB concentrations upon protein expression during this phase, which led to increased metabolic burden for the host (Figure 4B). Rise in IB concentrations was indicated by a change of the carbon dioxide to substrate yield (Y_CO2/S_ not shown). Phase II was characterized by constant yields and carbon balance (C-balances). This phase overlapped with phase III, where recovery of productivity set in. Acetate formation measured by photometric assays (not shown) was always low for all cultivations. In contrast, BBlue chemostat did not show a phase I increase in yield coefficients of the C-balance. We believe that this was due to lack of IB expression in this strain. Surprisingly, a drop in C-balance was present upon recovery of productivity at 100–150 h (phase II and III). C-balance did not close to 1 during the entire cultivation, which indicated some other non-measured metabolites during this run. Reduction of biomass formation (decreased μ) is a common phenomenon upon RPP (Neubauer et al., 2003; Hoffmann and Rinas, 2004). Effects could be clearly seen for *E. coli* expressing GFP and mCherry in the beginning and for BBlue at the start of the recovery phase (Figures 1, 3). These effects were obviously strongly dependent on the expressed protein and could not be generalized. Comparing results of the mCherry chemostat cultivation to a chemostat cultivation for production of mCherry in literature (Schmideder and Weuster-Botz, 2017), maximum specific titer was found to be higher by a factor of 100, whereas average specific titer was higher by a factor of 10 (Table 4). As referred chemostat cultivation was induced with IPTG, we suppose that lactose induction facilitated the production of mCherry in chemostat cultivation.

Higher dilution rates are beneficial for productivity in chemostat, as described in literature before (Seo and Bailey, 1986; Reinikainen and Virkajärvi, 1989). However, no discussion on long term stability was given, as generally steady state was assumed after three residence times (Seo and Bailey, 1986), which corresponds to *D* = 0.1 h^–1^ at 30 h. This would only result in 17.3 generation times at the presented *D* = 0.4 h^–1^, where maximum productivity was found. The cultivations presented here, were cultivated for almost 40 generations. Stability of the long-time process is the main concern using *E. coli* as host (Peebo and Neubauer, 2018). Genetic instabilities, like plasmid loss and mutations, happen during this long term of cultivation. The recovery of the productivity in phase III was a strong indicator that only minor plasmid loss happened during cultivations and that no plasmid deficient hosts overgrew the culture.

All chemostat cultivations except for the glycerol-lactose chemostat cultivation on Bl21(DE3) follow the often describe bell-shaped decrease of productivity (Peebo and Neubauer, 2018). It is not clear yet, why glucose based chemostats showed a complete loss of productivity and glycerol-based cultivations recovered after a certain time span. Diauxic growth is known to downregulate β-galactosidase activity, leading to inducer exclusion often observed in batch cultivation (Monod, 1949; Inada et al., 1996). As feeding of carbon source and inducer was conducted using previously established feeding protocols, growth rates never exceeded μ_max_ (Wurm et al., 2017a). Both carbon sources were taken up at all time, except for a monitored adaption phase observed for HMS174 (Supplementary Figure 1). Therefore, diauxic growth can be excluded as a possible explanation. Even though IPTG transport was found to be highly dependent on lactose permease (Fernández-Castané et al., 2012), chemostat cultivations with Bl21(DE3) and IPTG induction also resulted in an irreversible drop of productivity (Kopp et al., 2019a). Only galactose accumulation was found to alter when comparing glucose and glycerol cultivations on Bl21(DE3), which will be discussed in more detail. Acetate formation was present in HMS174 strains for both carbon source, while acetate concentration was always below limit of detection of our respective HPLC method for Bl21(DE3) strains (Supplementary Figure 1).

Our recent work on fed batch cultivations using mixed feeds systems with glucose/lactose and glycerol/lactose showed a strong increase on productivity upon glycerol feeding (Kopp et al., 2017). Higher and better performance of cultivations using glycerol as carbon source was also found to produce different enzymes (Hansen and Eriksen, 2007; Liu et al., 2011). Difference in enzymatic regulations on glycerol consumption were reported by activation of gluconeogenesis also including “carbon stress responses” for batch culture approaches (Martínez-Gómez et al., 2012). However, in the performed chemostat cultures this glycerol carbon stress could be clearly seen for *E. coli* expressing GFP (Table 2) in yields and productivity compared to the glucose/lactose mixed feed system. Results indicate that glucose is the carbon source of choice in the beginning of a chemostat culture, as higher productivity upon glucose cultivation can be observed. As μ_max_ is higher upon glucose cultivation compared to glycerol, energy (ATP) needed for recombinant protein formation might be faster accessible upon start of induction (Kopp et al., 2017; Wurm et al., 2017b).

The faster metabolism on glucose cultivation, compared to glycerol consumption (Wang and Yang, 2013) could possibly explain higher productivity for glucose fed chemostats in the beginning of the cultivation. NADPH formation on glycerol is also meager, when compared to glucose cultivation, emphasizing the previous statement (Yao et al., 2016). Still, in contrast to glucose, glycerol enabled a recovery of productivity at later stage. Reasons still need to be investigated but could possibly result from different intracellular metabolism (i.e., pyruvate dehydrogenase and TCA activity) (Murarka et al., 2008; Yao et al., 2016).

**FIGURE 5:**
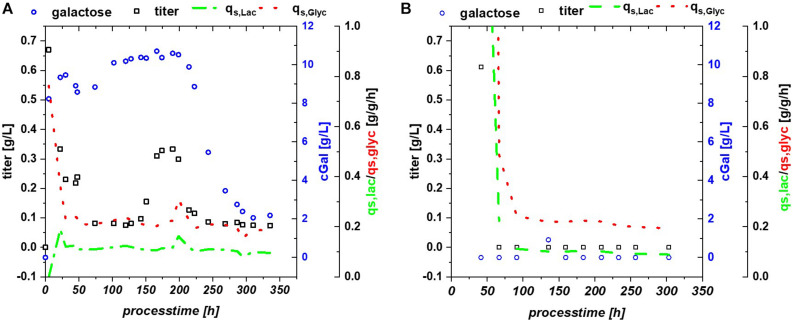
**(A)** Bl21(DE3) expressing GFP: glycerol/lactose cultivation: specific substrate uptake rates are constant throughout the whole induction time, while titer of the recombinant protein and extracellular galactose concentrations show high fluctuations; **(B)** HMS174 expressing GFP: glycerol/lactose cultivation with no accumulation of galactose and no respective recovery of productivity.

Multistability of induction was described by Ozbudak et al. (2004) showing changes in expression. In this publication, it is shown that the lac system can remain in several states, depending on the amount of LacI and cAMP molecules. With constant process parameters, changes in plasmid number during the cultivation might be a reasonable explanation for the observed effects. Based on the strong metabolic burden in the beginning, total number of plasmids might change and lead to a bi-stability of the cultivation system. Yet, pET plasmids generally carry a LacI gene, resulting in a direct relationship between lac repressor and plasmid number and may drastically reduce those observed effects during pET-based induction. Furthermore, the study by Ozbudak et al. (2004) was performed with a strong irreversible inducer [thiomethyl galactoside (TMG)], in comparison to lactose used for our experiments. Glycerol does not provoke a cAMP response directly, but cAMP response might be present based on the metabolized glucose (from lactose) as seen in fed batch cultivations (Kopp et al., 2017), which inherited a very different regulation compared to the glucose based cultivations. A further possible alternative for this fluctuation in productivity might be intracellular galactose accumulation (Supplementary Figure 1). Galactose is known to affect the cellular β-galactosidase concentrations (Llanes and McFall, 1969; Portaccio et al., 1998) and has been shown to function as a weak inducer (Ukkonen et al., 2013). High intracellular galactose concentration (drop in extracellular concentration), seen in Figure 5A, was accompanied by a drop of productivity. However, lactose consumption was not affected by the galactose fluctuations.

HMS174 can metabolize galactose upon induction with lactose (Hausjell et al., 2018), therefore this strain was investigated using a mixed feed with glycerol/lactose. However, previously mentioned advantages, such as overflow metabolism and overall titer formation favor B strains over K strains for the RPP (Shiloach and Fass, 2005; Rosano and Ceccarelli, 2014). HMS174(DE3) expressing GFP revealed no recovery on glycerol and no pronounced accumulation of neither glycerol, nor lactose or galactose, compare to Figure 5B. Still constant overflow metabolism was found for HMS174(DE3) chemostat cultivations whereas no acetate accumulation could be monitored for Bl21(DE3) (Supplementary Figure 1). As galactose was completely metabolized in HMS174(DE3) cultivations, biomass was found to be higher compared to Bl21(DE3) cultivations. Specific uptake rates were also constant, expect for deviations upon start of the induction phase. Yet, we believe that galactose variations are the most reasonable explanation for the effects seen in Bl21(DE3). It is not clear why galactose is stored inside the *E. coli* cells or expelled into the supernatant as seen in shifts of galactose concentration. Previous studies indicated galactose as main player for Lazarus effect. Based on these findings we expect a non-trivial interaction of multiple factors in Bl21 resulting in the observed behavior.

Furthermore, the three analyzed fluorescence proteins exhibit several differences. GFP originates from jellyfish *Aequorea Victoria* while mCherry from *Discosoma* sp. (Shaner et al., 2004) and BBlue from *Actinia equina*. BBlue and mCherry show a homology of about 60% identical amino acids. GFP was found to be highly different, as overlap in gene sequence is below 30% of amino acids. All three analyzed proteins do not have any disulfide bonding, predicted via Disulfind server (Ceroni et al., 2006). However, a high difference is reported in maturation kinetics. While GFP only exhibits one maturation step (oxidation), for mCherry a two-step mechanism is described. Therefore, far longer maturation times for mCherry, than for GFP about a factor of 10 were reported (Hebisch et al., 2013). Both maturation kinetics exhibit a pronounced growth rate dependency. Knowledge about the maturation may be a key parameter in further analysis of bacterial subpopulations during chemostat cultivations (Hebisch et al., 2013).

Finally, we compared time space yields for the three glycerol/lactose-based cultivations in Bl21(DE3) to fed batches in Table 5. No continuous glycerol/lactose cultivations could compete with fed batch cultivation. mCherry showed the highest time space yield in tested cultivations. It needed at least 167 h of cultivation with an averaged time space yield to meet the performance of a classic fed-batch (which exhibits about 7.5 g/l titer after 10 h of induction).

**TABLE 5:** Average time-space-yield (TSY) of fed batch and glycerol/lactose chemostat including running time of chemostat to reach the performance of the fed batch for BL21(DE3).

Protein	Glucose-IPTG fed-batch average TSY [g/l/h]	Glycerol-lactose chemostat TSY [g/l/h]	Cultivation time to reach fed-batch productivity at 10 h of induction [h]
GFP	1.28	0.022	581 h
mCherry	0.87	0.052	167 h
BBlue	0.56	0.017	215 h

Long-term *E. coli* cultivations are known to cause different mutants, showing different physiology (i.e., μ, Y_*X/S*_, etc.) compared to the original strain (Weikert et al., 1997; Binder et al., 2017; Delvigne et al., 2017; Heins et al., 2019). Long cultivation durations with wild-type strains revealed that changes in transcriptome and proteome can be already seen without recombinant protein formation (Wick et al., 2001; Peebo and Neubauer, 2018). As exact regulations in recombinant protein formation are still unknown, reasonable control strategies with further insight in the metabolic regulation are needed. We have ongoing projects identifying different productive subpopulations in *E. coli* being responsible for the change in productivity. However, new insights into the “black box” using transcriptomic analysis and measurement of plasmid and cAMP contents of the glycerol/lactose mixed feed system is mandatory and are ongoing in our group.

Summarizing, in this study we tested glycerol/lactose mixed feed systems in continuous culture using *E. coli* Bl21(DE3) and HMS174 expressing different model proteins to find a system with long time stable productivity potentially outperforming fed-batches. Beside lower productivity of glycerol/lactose chemostats compared to glucose/lactose in the beginning, a recovery of productivity at times larger than 100 h (about 15 generation times) could be observed for all strains investigated. However, further analysis like subpopulation monitoring is necessary to understand this “Lazarus” effect on a cellular level.

## Data Availability Statement

The original contributions presented in the study are included in the article/Supplementary Material, further inquiries can be directed to the corresponding author.

## Author Contributions

SK, JK, PV, and CS performed the bioreactor cultivations. SK, JK, and CS evaluated the data. CH, OS, and CS supervised the work. CH and FD gave valuable input for the manuscript. OS and CS drafted the manuscript. All authors contributed to the article and approved the submitted version.

## Conflict of Interest

The authors declare that the research was conducted in the absence of any commercial or financial relationships that could be construed as a potential conflict of interest.
