# Supplementary material for: The Lazarus Escherichia coli Effect: Recovery of Productivity on Glycerol/Lactose Mixed Feed in Continuous Biomanufacturing
Source: Front Bioeng Biotechnol. 2020 Aug 13;8:993. doi: 10.3389/fbioe.2020.00993 (PMC7438448; doi:10.3389/fbioe.2020.00993)
Supplement: Supplementary file 1 [file Image_1.pdf]

Supplementary Material

The Lazarus *Escherichia coli* effect: Recovery of productivity on glycerol/lactose mixed feed in continuous biomanufacturing

Stefan Kittler[2+], Julian Kopp[1+], Gwen Veelenturf[1], Oliver Spadiut[2], Frank Delvigne[3], Christoph Herwig[1,2] and Christoph Slouka[2*]

+These authors contributed equally to the work.

[1]Christian Doppler Laboratory for Mechanistic and Physiological Methods for Improved Bioprocesses, Institute of Chemical Environmental and Bioscience Engineering, Vienna University of Technology, Vienna, Austria

[2]Research Division Biochemical Engineering, Institute of Chemical Environmental and Bioscience Engineering, research group Integrated Bioprocess Development, Vienna University of Technology, Vienna, Austria

[3]Université de Liège - Gembloux Agro-Bio Tech TERRA research and teaching centre Microbial Processes and Interactions (MiPI) Avenue de la Faculté, 2B, B-5030 Gembloux, BELGIUM

*** Correspondence:**
Christoph Slouka.
christoph.slouka@tuwien.ac.at

Keywords: ***E. coli*, recombinant protein production, continuous biomanufacturing, mixed-feeding, productivity recovery (Min.5-Max. 8)**

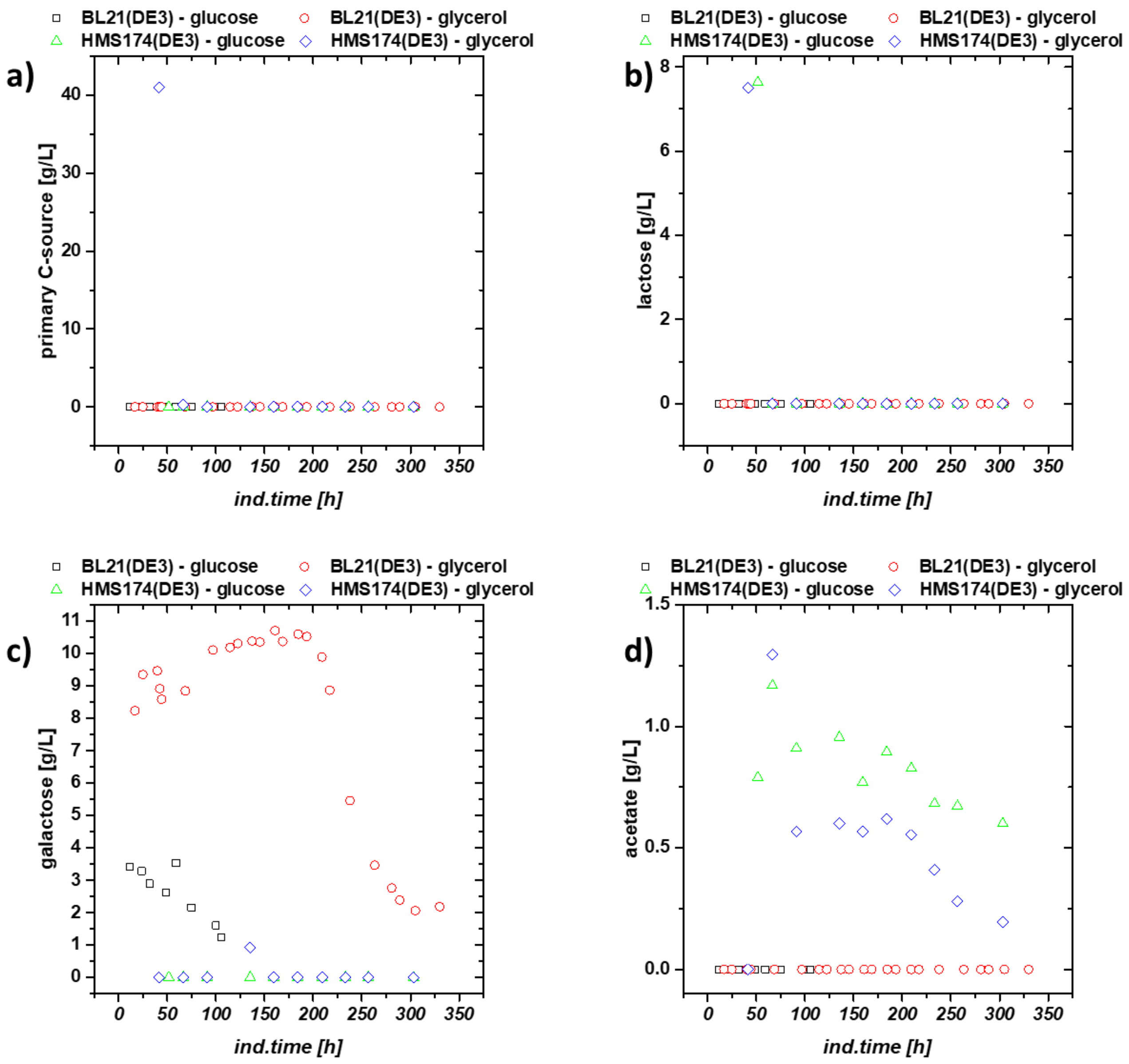

Supplementary figure 1: showing sugar accumulation measured in the fermentation broth for all chemostat cultivations conducted for the target protein GFP. Cultivations vary in primary C-source (i.e. glucose or glycerol) and host strain (i.e. BL21(DE3) or HMS174(DE3)).

Sugar accumulation is given in (a) for the primary C-source (glucose or glycerol), (b) the inducer (lactose), (c) galactose accumulation and (d) acetate accumulation

It can be shown that inducer and carbon source are taken up at all time, except for the adaption phase monitored for HMS174(DE3) cultivations. We hypothesize that this adaption phase occurs due to lower metabolism for HMS174(DE3). Galactose was found to accumulate in fluctuating amounts for BL21(DE3), possibly declaring shifts upon productivity, as emphasized at later stage in the discussion. Acetate was found to accumulate in HMS174(DE3) cultivations, whereas no acetate was found for BL21(DE3) cultivations. Monitored overflow metabolism for HMS174(DE3) is in accordance with literature.
